# Lactopeptine

**Published:** 1879-07

**Authors:** 


We learn from all source that the Lactopeptine of the New York Pharmacal Association is proving a most efficient agent in the treatment of all bowel troubles, and that its power is most clearly recognized in the treatment of cholera infantum. It is a standard preparation and worthy of a trial.—*Nashville Journal of Medicine and Surgery.*

